# A global conversation about energy from biomass: the continental conventions of the global sustainable bioenergy project

**DOI:** 10.1098/rsfs.2010.0047

**Published:** 2011-02-02

**Authors:** Lee Rybeck Lynd, Ramlan Abdul Aziz, Carlos Henrique de Brito Cruz, Annie Fabian Abel Chimphango, Luis Augusto Barbosa Cortez, Andre Faaij, Nathanael Greene, Martin Keller, Patricia Osseweijer, Tom L. Richard, John Sheehan, Archana Chugh, Luuk van der Wielen, Jeremy Woods, Willem Heber van Zyl

**Affiliations:** 1 Thayer School of Engineering, Dartmouth College, Hanover, USA; 2 Institute of Bioproduct Development, Universiti Teknologi, Johore Bahru, Malaysia; 3 São Paulo Research Foundation, FAPESP, São Paulo, Brazil; 4 Department of Process Engineering, University of Stellenbosch, Stellenbosch, South Africa; 5 University of Campinas, Campinas, Brazil; 6 Department of Science, Technology and Society, Utrecht University, Utrecht, The Netherlands; 7 Natural Resources Defense Council, New York, NY, USA; 8 Oak Ridge National Laboratory, Oak Ridge, TN, USA; 9 Department of Biotechnology, Delft University of Technology, Delft, The Netherlands; 10 Department of Agricultural and Biological Engineering, Pennsylvania State University, University Park, PA, USA; 11 Institute of the Environment, University of Minnesota, St. Paul, MN, USA; 12 School of Biological Sciences, Indian Institute of Technology Delhi, Delhi, India; 13 Centre for Environmental Policy, Imperial College London, London, UK; 14 Department of Microbiology, University of Stellenbosch, Stellenbosch, South Africa

**Keywords:** plant biomass, bioenergy, global sustainable bioenergy project

## Abstract

The global sustainable bioenergy (GSB) project was formed in 2009 with the goal of providing guidance with respect to the feasibility and desirability of sustainable, bioenergy-intensive futures. Stage 1 of this project held conventions with a largely common format on each of the world's continents, was completed in 2010, and is described in this paper. Attended by over 400 persons, the five continental conventions featured presentations, breakout sessions, and drafting of resolutions that were unanimously passed by attendees. The resolutions highlight the potential of bioenergy to make a large energy supply contribution while honouring other priorities, acknowledge the breadth and complexity of bioenergy applications as well as the need to take a systemic approach, and attest to substantial intra- and inter-continental diversity with respect to needs, opportunities, constraints and current practice relevant to bioenergy. The following interim recommendations based on stage 1 GSB activities are offered:

— Realize that it may be more productive, and also more correct, to view the seemingly divergent assessments of bioenergy as answers to two different questions rather than the same question. Viewed in this light, there is considerably more scope for reconciliation than might first be apparent, and it is possible to be informed rather than paralysed by divergent assessments.
— Develop established and advanced bioenergy technologies such that each contributes to the other's success. That is, support and deploy in the near-term meritorious, established technologies in ways that enhance rather than impede deployment of advanced technologies, and support and deploy advanced technologies in ways that expand rather than contract opportunities for early adopters and investors.
— Be clear in formulating policies what mix of objectives are being targeted, measure the results of these policies against these objectives and beware of unintended consequences.
— Undertake further exploration of land efficiency levers and visions for multiply-beneficial bioenergy deployment. This should be unconstrained by current practices, since we cannot hope to achieve a sustainable and a secure future by continuing the practices that have led to the unsustainable and insecure present. It should also be approached from a global perspective, based on the best science available, and consider the diverse realities, constraints, needs and opportunities extant in different regions of the world.

The future trajectory of the GSB project is also briefly considered.

## 1. Introduction

Plant biomass is one of a limited set of options as the world looks to become less reliant on non-renewable fossil resources. In evaluating this alternative, recent assessments of the feasibility and desirability of bioenergy (fuels and electricity) have been sharply divergent, and indeed exhibit a bimodal distribution, with most studies envisioning either a very large or a very small role for biomass in energy supply [1,2]. The seemingly contradictory claims about the feasibility and desirability of large-scale bioenergy production are understandably confusing to policy-makers, impede effective action, and are unacceptable in light of the urgency of energy and sustainability challenges facing humanity.

The situation is admittedly complex, in part because of the substantial number of interconnected issues bioenergy production impacts and is impacted by. Concern is widespread over potential conflicts of bioenergy production with food supply [3] and indirect land use [4,5]. Some crops may increase erosion, degrade soil and water quality, and reduce biodiversity, while others are likely to have much more positive effects. In particular, some cropping systems may incur large carbon debts through land-use change and high emissions through intensive management, while others can sequester carbon while simultaneously producing large quantities of fuel [4,6]. Similarly, the social, cultural and economic dimensions of a rapidly growing industrial sector could follow different trajectories, seeding either many new independent businesses or a few large international companies, with corresponding impacts on community well being [7].

The global sustainable bioenergy (GSB) project was initiated in 2009 by a group of scientists, engineers and policy experts from universities, government agencies and the non-profit sector from across the globe, with the overall goal of providing guidance with respect to the feasibility and desirability of sustainable, bioenergy-intensive futures. In the summer of 2009, a statement on behalf of GSB project organizers observed [8]:

Although there is a natural reluctance to consider change, we must do so, because humanity cannot expect to achieve a sustainable and secure future by continuing the practices that have resulted in the unsustainable and insecure present.

Consistent with this perspective, the GSB project seeks to take a different approach from many other worthy initiatives in the bioenergy field. Rather than focusing on what is most probable, the GSB project is focused on what is most desirable. Rather than reflecting often sharply divided expert opinion, the GSB project seeks to build new understanding and consensus. Rather than having the present as a point of reference, the point of reference for the GSB project is a vision for the future.

The project is structured in three stages:

*Stage 1*. Hold five continental conventions with outcomes as follows:

a. endorse a common resolution about the importance of bioenergy and the goals of the GSB project;
b. gather input on structuring the analysis to be carried out in stages 2 and 3;
c. approve resolutions representing perspectives on bioenergy, including key questions and opportunities, from each of the world's continents;
d. write a report encompassing a, b and c;
e. recruit participants and support for stages 2 and 3.

*Stage 2*. Explore whether and how it is physically possible for bioenergy to sustainably meet a substantial fraction of future demand for energy services—e.g. 150 EJ annually,^1^ corresponding to the 23 per cent of primary energy supply expected from biomass in the IEA Blue Map Scenario [10]—while feeding humanity and meeting other needs from managed lands, preserving wildlife habitat and maintaining environmental quality.

*Stage 3*. Analyse and recommend transition paths and policies in light of stage 2 results, incorporating analysis of macroeconomic, environmental, ethical and equity issues as well as local-scale effects on rural economies.

Data for the five continental conventions held during 2010 are summarized in table 1. The full text of the five continental conventions may be found online (http://engineering.dartmouth.edu/gsbproject/). This paper describes what took place at the stage 1 continental conventions, what was learned and the future direction of the GSB project.

**Table 1.** Summary data for the stage 1 continental conventions of the global sustainable bioenergy project.

continent	location	dates (2010)	host institution	chairs	sponsors	number of attendees
Europe	Delft, The Netherlands	26–26 Feb	Kluyver Centre for Industrial Fermentations	Patricia Osseweijer, Andre Faaij	Kluyver Centre for Genomics of Industrial Fermentation, Netherlands Organisation for Scientific Research; Delft University of Technology	70
Africa	Stellenbosch, South Africa	17–19 Mar	University of Stellenbosch, South Africa	Emile van Zyl	SANERI Chair of Biofuels; Stellenbosch University; National Research Foundation	40
Latin America	Sao Paulo, Brazil	23–25 Mar	The São Paulo Research Foundation (FAPESP)	Brito Cruz, Jose Goldemberg	FAPESP Bioenergy Research Program (BIOEN), Brazilian Academy of Sciences (ABC)	200
Asia, Oceania	Kuala Lumpur, Malaysia	14–16 Jun	Universiti Teknologi Malaysia	Ramlan Aziz	Ministry of Energy, Green Tech & Water	86
North America	Minneapolis St Paul, USA	14–16 Sep	University of Minnesota	John Sheehan, Jon Foley	Institute for Renewable Energy and the Environment	64

## 2. Summary of the stage 1 conventions and themes of the continental resolutions

### 2.1. Europe

The first Continental Convention took place in Delft, The Netherlands, in February 2010. Some 70 participants from 10 European countries and four other continents gathered to discuss the two main questions of the GSB project, i.e. ‘Can we produce enough biomass to substantially contribute to our energy needs in a sustainable way?’ and ‘Do we need to, or are there other, better, technologies available to sustainably meet our future energy needs?’.

The meeting brought together leading experts from multiple fields and organizations, including academics, industrialists, NGO representatives and policy experts from, for example, the Organisation for Economic Co-operation and Development (OECD) and the International Energy Agency. Enriched by this diversity of perspectives, in-depth discussions occurred and information and perspectives were exchanged, leading to increased understanding of current practices in Europe, the potential of a bio-based society and paths towards that end. This included views on agricultural practice and policy, nutrient recycling, biodiversity preservation, climate change issues, sustainability modelling, integrated assessment and economic modelling, potential of novel technologies involving biotechnology, biorefining, gasification and related areas.

#### 2.1.1. Conclusions from the European convention

It was established that Europeans aim to take a global lead with knowledge providing a powerful asset in balancing resource exploitation and behavioural change. They are used to a high standard of living with well-developed notions of sustainability, equity and social justice. Their ideal world view brings economic and environmental stability, measures to mitigate climate change, energy and food security and food safety for all.

The following drivers for developing bioenergy were identified:

— energy security (related to depletion of oil but especially dependence on oil- and gas-producing countries),
— economic security (employment and economic opportunities, especially in relation to the recent credit crunch),
— climate change, sound management of natural resources and overall sustainability, and
— agricultural economy and rural development.

Constraints were seen in disparities in resources (investment, land and waste), political opposition and lack of organized constituency and the challenge of producing biofuels in Europe at costs competitive with imports. Furthermore, participants agreed that while Europe has a strong vision on socio-ecological and justice measures, scientific uncertainty on sustainability issues and a consequent lack of clear criteria for certification brings these issues to the forefront of policy debate. From the perspective of bioenergy development, the European Commission and Parliament have focused on the need to develop and deploy sustainability criteria for biofuels and bioelectricity. Its Renewable Energy Directive is one of the leading policy frameworks that illustrate the attempt to reconcile energy, food and climate change security. Further development of such frameworks and experience with their deployment will continue to build up in the coming years. Nevertheless, many challenges lie ahead, especially developing and implementing holistic policy strategies that combine targets for renewable energy, rural development and sustainable land use and agriculture.

Public opinion was considered a potential problem with the negative European views on genetically modified organisms and the present negative food–fuel debate. These factors considerably decrease the political will to drive a bioenergy agenda. At the same time, the potential for biomass production in Europe was certainly recognized with the remark that there are considerable variations in regional capacity, with quite a potential in the Eastern countries such as Poland and Ukraine [11]. The strong knowledge infrastructure and proven tech-transfer practices should make it possible for Europe to drive the development of novel technologies, while the well-developed markets for both import and export provides another added value. Participants also felt that citizens in general are aware of a needed change in energy consumption and supply. In addition, Europe can provide opportunities for developing ‘sustainable intensification’ of agriculture and cropping systems [12].

Participants concluded that Europe has a stable population, with a stable energy demand, but also with strong regional differences. They also stated that the present energy portfolio is *not* secure, *nor* sustainable. Furthermore it is probable that Europe will be a net importer of bioenergy, which will bring issues of security, logistics, trade, sustainability and certification. Europeans see the development of second generation biofuels and biorefining as very relevant. Consumption patterns and understanding of public perceptions and behaviour are seen as challenges that needs to be addressed with public participation towards the transition to a bio-based agenda. These factors are also seen as critically important if bioenergy is to play a substantive role in the future.

The European resolution included the following statements: ‘Europe has the ability to provide substantial shares of its future energy demands from sustainable bioenergy. It has a unique set of opportunities … . to aggressively develop bioenergy solutions’, respecting food security and increasing sustainability; recognizing regional opportunities and involving stakeholders'. It was estimated that 40 million hectares will be available to sustainably produce bioenergy from which 30 per cent of Europe's primary energy needs can be produced [11].

### 2.2. Africa

The second convention was held in South Africa, from 17 to 19 March 2010. Like the European convention, the African convention brought participants with a diversity of backgrounds and priorities. The geographical potential of Africa to produce plant biomass is at least as large as any other continent and far exceeds the requirements for food and basic needs for the African population. Yet, a vision for bioenergy responsive to African challenges is not well-developed, and is both an urgent need and an opportunity. Prominent themes of the discussion included the different circumstances in Africa relative to other continents and the need for bioenergy to respond to pressing African challenges associated with an interconnected set of issues involving poverty, food security, economic development, gender issues, health and energy security.

Valuable ‘lessons’ were identified for further research and action.

#### 2.2.1. Lesson 1

The success of bioenergy in Africa depends on the extent to which it will contribute towards meeting the critical socio-economic needs of the African population. These socio-economic needs include food and household energy security, health, job creation and gender development. For instance, in many African households, women and girls take the burden of providing energy for cooking and heating. Such responsibility takes away valuable time of these women and girls from participating in activities of high economic value such as education. Therefore, implementation of bioenergy systems should relieve women and girls from the burden of fetching firewood.

#### 2.2.2. Lesson 2

Most African countries, such as Malawi, Madagascar, Zambia, Mozambique, Tanzania and Democratic Republic of Congo, have less than 10 per cent of the population with access to electricity. Consequently, over 80 per cent of the total energy supply for heating, cooking, production and processing of agricultural produce is derived from biomass, such as fuel wood and agricultural residues. Despite the availability of biomass and existence of local knowledge in the production, conversion and utilization of bioenergy, there is a lack of adequate capacity to develop economically viable and sustainable bioenergy systems.

The traditional conversion of biomass into energy is often considered to be less sustainable because it is extractive and relies on inefficient technology. As a result, the full economic potential of the feedstocks is not realized. Therefore, there is a need for the introduction of improved technologies for production, conversion and utilization of biomass in order to increase the value generation and retention. Such advanced bioenergy technologies should provide poor communities with the products and energy services that reduce the workload of women and girls in gathering fuel wood and slow down or eliminate the over-exploitation of forests. In addition, there is need for targeted education to increase local expertise and knowledge coupled to the provision of support services such as extension services and provision of subsidies in agricultural inputs, which has shown to be successful in countries such as Malawi.

#### 2.2.3. Lesson 3

Improvements in small-scale agricultural production are a prerequisite for the success of bio-energy in Africa. This is because most African countries are agro-based and dominated mainly by subsistence farmers. Bioenergy production should be viewed as a means for diversifying agricultural production that would provide rural communities of Africa with new markets and additional sources of income. However, the policies in agriculture, energy supply, forestry, local and international trade, rural development, environment, land tenure and capacity building (education) do not always complement each other. Therefore, implementation of sustainable bioenergy systems will require integration and harmonization of these policies. At national and regional levels, these bioenergy systems should provide a platform for expanding initiatives in a multi-functional agriculture system that serves multiple bio-based industries and rural development through provision of necessary infrastructure such as markets and roads.

#### 2.2.4. Lesson 4

Compatibility of bioenergy systems with food security in Africa lies in choosing appropriate system boundary choices such as:

— Careful selection of combination of crops so that they are culturally and environmentally acceptable while not worsening, and ideally enhancing, food security.
— Improvements in land use and crop husbandry practices.
— Provision of agricultural support services such as extension and subsidies in the form of starter packs.
— Market access.
— Food and energy balances.
— Training in agro-forestry and value adding (processing) of agriculture and forestry products.
— Ownership of land and resource allocation by considering implementation models, which are inclusive of all stakeholders to ensure ownership and avoid marginalization of the African poor.
— Political will and buy-in from all stakeholders, including political leaders.

#### 2.2.5. Lesson 5

The bioenergy systems depend on the availability of forests or idle agricultural or pasture land in Africa, which have important economic, environmental and cultural roles in African communities. However, the introduction of advanced bioenergy systems cannot completely replace the traditional bioenergy systems. Therefore, bioenergy systems should be pro-poor, implemented in ways that allow the African population to access both traditional and advanced sources of biomass energy.

#### 2.2.6. Lesson 6

Much of the benefit from implementation of advanced bioenergy systems can be realized through the accompanying investment in land, infrastructure and human resources. This subsequently will increase agricultural productivity and protection of the environment in Africa, while inclusive bioenergy systems would provide both on-farm and off-farm sources of income throughout the value chain and encourage equitable modus operandi. Some African governments are already pro-active and strongly committed to developing innovative policies and supporting programmes for bioenergy. For instance, countries such as Malawi and Zimbabwe produce ethanol that is blended in transportation fuel.

A fresh look at the role that bioenergy can play in the future of Africa resonated in the opening statement of the African continental resolution: as the world considers paths to a sustainable future and the role of bioenergy in this context, Africa brings important assets and wants to be an active partner but needs to ensure that bioenergy development is implemented in a way that contributes to critical human needs. A sustainable globe requires a sustainable Africa.

### 2.3. Latin America

The third Convention was held in Sao Paulo, Brazil from 23 to 25 March 2010 and was attended by approximately 200 researchers, mostly from Brazil, but with some representatives from Argentina and Mexico [13]. Presentations primarily addressed the Brazilian experience with sugarcane ethanol, with a focus on topics related to the sustainability of large-scale use of ethanol, but some presenters discussed other perspectives for Latin America, including the Argentinean programme for biodiesel.

Clearly the Brazilian experience is inspiring for the region, especially for the countries that have land and climate adequate for growing sugarcane. Latin America has land, favourable climate, diverse feedstock options and technology that has been deployed regionally and could be expanded continentally in a sustainable manner without compromising food security and ecosystems. According to Doornbosch & Steenblik [14], of the total world availability of land to produce bioenergy expected by 2050 (440 Mha), around 60 per cent (250 Mha) will be in Latin America, mostly in Brazil, Colombia, Bolivia and Argentina.

An important characteristic of sugarcane ethanol is that it displays advanced biofuel performance while at the same time requiring simple technology for its production. This is especially interesting for developing countries.

The region has proven potential to fulfil an important role in providing biofuel for local as well as world demand. Latin America alone produces around 30 billion litres of bioethanol and about 10 billion litres of biodiesel, representing 40 per cent of bioethanol and 20 per cent of biodiesel produced in the world. The main bioenergy producing countries in Latin America are: Brazil, Argentina, Colombia and Mexico. Historically biofuel production in Latin America began as a necessity in response to energy security concerns, but has progressed into an opportunity for economic and social development.

Latin America has implemented a biorefinery model with increasingly integrated feedstock crop systems, co-products and a large reduction of green house gas emissions.

Bioenergy is being produced in Latin America with fair sustainability indicators. Biofuels are produced without subsidies, with increasing yields and dropping cost. In many cases it has been demonstrated that biofuels help to promote rural economic development [15] and creates synergies with production of food such as sugar, for which Brazil is a world leader.

There are at least two successful cases where bioenergy has proved sustainable and enabled development. These are biodiesel production in Argentina and sugarcane ethanol in Brazil. The Brazilian case has demonstrated the feasibility of large-scale production: 18 per cent of the total primary energy supply in the country comes from sugarcane [16] and sugarcane ethanol substitutes for more than 50 per cent of the gasoline that would otherwise be used [17], making sugarcane the second most important energy source in the country, following oil and ahead of hydroelectricity and traditional biomass. Legislation is in place, and increasingly effective, that establishes environmentally sound agro-ecological zoning, reflecting widespread consensus among policy-makers and the general public on the importance of preserving biodiversity.

Recent studies have shown that the region presents an enormous potential to become a net bioenergy exporter. According to Cerqueira Leite *et al.* [18], Brazil alone could replace 5 per cent of world demand of gasoline by 2025 using only 4 per cent of its territory and without jeopardizing its status as a world food supplier nor endangering its eco-sanctuaries such as the Amazon, Pantanal or the Atlantic Rainforest. According to an encompassing agro-ecological zoning study for sugarcane conducted by the Brazilian Ministry of Agriculture, there are about 60 Mha of available land presently occupied with degraded pasture that can be used for sugarcane with no significant impact on environment and biodiversity.

The participants of the São Paulo GSB Convention concluded that expanded realization of societal benefits from bioenergy production in Latin America would be fostered by:

— Government support to normalize common policies, such as certification for sustainability and blends.
— Development of new technologies that can process a variety of feedstocks at a variety of scales and are responsive to local circumstances, improve each link in the supply chain, and allow flexible production of complementary co-products.
— Increased understanding and consensus with respect to sustainability issues involving science, government and broader society.
— An agenda for R&D and for human resource development that can help countries deal with the rapid technological advances.

### 2.4. Asia–Oceania

The Asia–Oceania Convention was held in Kuala Lumpur, Malaysia, from 14 to 16 June 2010. The convention was represented from the member countries such as Malaysia, India, China, Indonesia, Thailand and Australia. Participants from three other continents (Europe, North America and Latin America) also attended this convention. The convention shared general perspectives of the GSB project and focused on the Asia–Oceania needs and priorities keeping in sight the huge regional diversity within the continent in terms of climate, geography, biological resources, cultural traditions and politico-economic situations. A range of biomass feedstocks are employed for bioenergy production in the Asia–Oceania countries, such as oil palm (Malaysia and Indonesia), Jatropha (Laos, Vietnam and China), Sugarcane molasses (India), Cassava (Thailand and Laos), Rice (China) and Eucalyptus (Australia) for biofuel production. In addition, many countries have defined policies and targets in anticipation of second generation biofuels. The various constraints, solutions and opportunities for developing ‘bioenergy’ as a viable, economical and hence sustainable industrial sector were explored contributing towards the Asia–Oceania vision for bioenergy in a holistic manner.

Asia–Oceania is a huge area representing about half the world's population, and while diversity of perspective was mentioned in connection with many continents, nowhere is this more evident than for Asia–Oceania. In part as a result, a continental perspective and identity is perhaps less-developed for many parts of Asia as compared with the other continents. Asia has seen and will continue to see fast economic growth in the present decade and increasing energy demand. There is a rapidly growing interest in the development of renewable energy programmes and policies on the part of national governments as well as industry-academia with the overall goals of achieving energy security, independence and sustainable development. Asia–Oceania harbours great biomass production potential with varied geo-political attributes promoting the interest of member nations to use bioenergy as a major renewable energy resource. Countries such as Japan, India, Malaysia and China already have well-established alternative energy programmes and policies.

Attendees recognized and highlighted the importance of developing bioenergy in ways that preserve Asia's unique biodiversity. There was widespread agreement that this can be done, but that it will require diligent attention and cannot be taken for granted. There was also consensus that development of skilled labour and novel technologies are needed to properly manage and use the wide range of biomass available in the biologically diverse Asiatic countries.

Emerging economies from the Asiatic region have an urgent need to meet rapidly growing energy demand, and that demand cannot be fulfilled sustainably by conventional fuels. Biomass-derived energy can provide not only energy security, price stability and a healthy environment, but also has the potential to promote rural prosperity, employment and infrastructure development. The convention participants proposed development of a biofuel industry in the Asia–Oceania region using inputs from both traditional knowledge and academic collaborations, strengthening research and development, technology transfer and adaptations both intra- and inter-continentally. The building of a robust bioenergy intellectual property portfolio in the region will attract the financial investments and interest of the corporate sector, encouraging corporate social responsibility and market demand owing to increasing concern among public for clean, green technologies and products.

There is strong evidence that bioenergy production in the Asia–Oceania region can contribute to energy security not only in its region but also participate at the global level owing to the richness of its various resources and technical capabilities. Development of biomass inventories and their assessment, agro-ecological zoning for conservation of biodiversity and judicial use of resource for bioenergy production are some of the initial action plans discussed at the meeting. There was also consensus that knowledge development and public awareness are important components for the success of the biofuel sector in the Asia–Oceania region. Gender role assessment and sharing of the benefits with the traditional knowledge stakeholders will aid in promoting the growth of the sector in the Asia–Oceania region at all levels of society. There was also support for the development of a harmonized framework that promotes the biobased economy, trade regulation to overcoming non-trade barriers, and climate negotiation. Consistently strong support from national governments as well as external agencies will further help in achieving the vision of a sustainable bioenergy future for Asia and Oceania.

### 2.5. North America

The GSB project held the last of its continental conventions in North America with discussants from the USA and Canada. Sixty representatives from industry, academia, NGOs and government met in Minneapolis, MN, to explore the challenges and opportunities of biofuels from a North American and a global perspective. This convention had the advantage of being informed by the findings of all the other discussions that occurred around the globe. It also occurred at a time of long and extensive policy dialogue in the USA about the impacts of renewable fuel policies being considered by the US EPA and the California EPA.

The North American stakeholders at the meeting brought a wide range of perspectives—from the cautious but hopeful perspective of environmental organizations seeking to avoid unintended consequences from biofuels to advocates for food security who identified rising prosperity in developing nations as a new and growing pressure on our global agricultural lands. Different perspectives were also apparent between stakeholders from the USA and Canada. Chief among these is the difference in attitudes between the USA and Canadian citizens on the relative importance of climate change. These differences led to different priorities for policy-makers in each country. Canadians see the potential for forest resources to greatly reduce their reliance on carbon intensive coal resources for heat and power. The USA is more focused on technology strategies that reduce dependence on foreign oil and provide opportunities for US farmers. These differences are made even more evident by the fact that Canada is a large exporter of petroleum to the USA.

#### 2.5.1. Conclusions from the North American convention

Building off the findings and resolutions of the previous conventions, participants forged and adopted a unanimous vision and approach to bioenergy in North America that is aggressive and comprehensive in nature. Their resolution asserts that ‘ … it is reasonable for North America to set a goal of producing 25% of our energy services from … bioenergy resources by 2050, and even greater contributions in the future’. But this can and must be achieved, according to the participants, in a way that holistically manages our land, water and other natural resources to increase bioenergy production without shortchanging growing food, feed and fibre needs and protecting the ability to provide valuable ecosystem services.

Stakeholders called for a strategy that includes:

— policies that encourage public and private investments in sustainable bioenergy technology,
— collection of ‘validated quantitative data to better guide policy development and business case development’,
— education, outreach and continued dialogue,
— support of holistic demonstrations that assess the impacts of bioenergy on all aspects of a sustainable society—social, environmental and economic, and
— continued measurement and monitoring to assure a sustainable direction for bioenergy.

The resolution declared ‘North America has benefited greatly from its endowment of fossil energy resources. We have both the capacity and the obligation to participate vigorously in the global transition toward a prosperous and sustainable future involving large scale bioenergy’.

## 3. Pan-continental perspectives and reconciliation of divergent assessments

The GSB continental conventions were instructive with respect to the breadth and complexity of the bioenergy field, challenges resulting from these features, and potential solutions to these challenges. It was evident that bioenergy spans a range of technologies and feedstocks from those currently deployed to those whose deployment is foreseen in the future, often with substantial performance improvements anticipated. In addition, bioenergy is potentially responsive to multiple motivations including rural economic development, energy security, enhancing ecosystem resilience, managing the impacts of conventional agriculture, improved balance of payments and large-scale sustainable energy supply. Finally, analysts commonly consider bioenergy in a range of time horizons and with a range of expectations for what could be accomplished in the future. The diversity of technologies, feedstocks, motivations and time horizons associated with bioenergy can legitimately be seen as desirable, but it also complicates assessment and policy formulation.

In light of this complexity, GSB participants challenged each other to think and communicate precisely about bioenergy as a means to promote development of widely shared and coherent understanding. Thus, for example, confusion can be avoided by being clear whether an analysis is based on certain specific technologies or all technologies, certain specific feedstocks or all feedstocks, and near-term or long-term time horizons. It was observed on multiple occasions that you ‘get what you incentivize’ in the policy domain. As a result, policies should ideally target desired outcomes, for example, greenhouse gas emissions, or avoidance of land clearing. Further, an action motivated by policies targeting one outcome may or may not serve other desired outcomes, for example, a policy aimed at promoting rural economic development may or may not foster sustainability or large-scale bioenergy production. Optimizing multiple criteria is difficult in such a complex interconnected system, so well-defined objectives, appropriate metrics and careful, comprehensive analyses will be critical if bioenergy policies are to achieve societal goals.

Part of the framework for discussion at the GSB conventions were the ‘*Can we?*’ and ‘*Must we?*’ questions, that is: *Can we* produce bioenergy at a very large scale while not sacrificing other important priorities? and *Must we* do so in order to have a reasonable expectation of achieving a sustainable world? [19]. As represented in the continental resolutions, there was widespread support among convention attendees for positive answers to both these questions. At the same time, there was a common view that there is a great divergence of opinion with respect to these questions, and that addressing this divergence is an urgent need. It was noted that it would be useful to understand how it is that presumably reasonable people with access to the same information can reach such apparently different conclusions.

In the course of the GSB conventions, the idea emerged that divergent assessments of bioenergy are often responses to different questions. Many analyses ask, in essence:

1. ‘What would be the impacts of large-scale use of current bioenergy technologies in a future world based on extrapolating current trends?’, while others ask:
2. ‘What role would bioenergy play in a future world reconfigured to meet energy and sustainability challenges?’.

Negative assessments commonly (although not always) result from asking question 1, and positive assessments commonly (although not always) result from asking question 2. Both questions have instructive answers, and both also have limitations. The biggest limitation of question 1 is that it does not illuminate paths to a sustainable world. The biggest limitation of question 2 is that it is not consistent with current trends. There is no logical contradiction in reaching different answers to these questions.

A major focus of the GSB conventions was brainstorming to identify behavioural or technological ‘levers’ that would allow large scale, sustainable production of bioenergy feedstocks from currently managed lands—including mobilization of biomass residues and waste streams as well as utilization of marginal lands—while honouring other priorities such as feeding the world and maintaining or enhancing environmental quality. Several such levers were identified, including but not limited to pasture intensification, land-efficient human diet and animal feed rations, winter double crops—perhaps in conjunction with protein recovery, and cultivation of water-efficient crops on semi-arid land. At each GSB convention, we observed a consensus that these and similar levers have great and relatively unexplored potential and merit further analysis. Investigating concrete implementation in different settings and regions is a key priority of phase 3 of the GSB initiative. Equally clear was the view that there is both a need and an opportunity to develop regionally responsive and informed visions for multiply-beneficial bioenergy deployment.

It was abundantly clear from the GSB conventions that the interplay of biophysical with economic and cultural resources will result in different trajectories as well as endpoints of bioenergy development. In areas where land is scarce and valuable, sustainable biomass may require intensifying agricultural production in ecologically responsible ways, while in other places it will be appropriate to develop low-input, extensive biomass production systems. In many regions, the greatest benefits from bioenergy, both in terms of reduced CO_2_ emissions and improved human health, will come from more efficient cookstoves or reducing reliance on electricity from coal. Even within a region, different sectors of society will have different financial resources and energy needs. Thus, we should encourage policies and technologies that allow for diverse and flexible solutions for biomass to satisfy local energy needs.

Many analyses projecting a large role for biomass energy, including most of those presented at the GSB conventions, anticipate new biomass production systems and conversion technologies. Discussions at the conventions frequently recognized the need to employ meritorious, current bioenergy technologies in ways that enable rather than impede deployment of future technologies, and to develop and deploy future processes in ways that expand rather than contract opportunities for early adopters and investors. At the same time, the common terminology of first, second, third and fourth generation biofuels implies a progression that will not and should not occur in all parts of the world. Sugar cane, annual grains, cellulosic feedstocks and algae may each play a distinct and complementary role, and the emergence of new technologies does not mean older technologies will not continue to advance.

There has been a widespread, and indeed nearly universal, assumption that increased reliance on biomass for energy will necessarily reduce the availability of land for crops, and thus increase the cost and scarcity of food. One of the most exciting ideas to take root during the GSB conventions was that bioenergy need not necessarily compromise food security, and indeed could enhance it. In particular, bioenergy has potential to positively impact key causes of food insecurity in the developing world—poverty, poorly developed infrastructure, agricultural export and aid policies in developed countries, and degraded land [20]. Further exploration of ‘win–win’ scenarios in which both energy security and food security are enhanced by bioenergy production is expected to be a focus of the GSB's activities going forward.

An immediate task of the GSB initiative is to develop the more detailed studies needed to confirm these regional opportunities, as well as the specific needs and the most optimal ways to achieve them. In turn, these regional visions and supporting analysis need to be communicated widely to gain further awareness and inform stakeholders. Several organizations, including the Brazilian Sao Paulo Research Foundation Fapesp, the Bio-based Ecologically Balanced Sustainable Industrial Chemistry consortium in The Netherlands, US Department of Energy Oak Ridge National Laboratory and several universities have begun to coordinate their research programmes to address these common goals. The GSB initiative is also formalizing a board to support these regional studies and to ensure a coordinated effort worldwide. This governance is intended to be open, transparent and collaborative, and other organizations that share the GSB goals are invited to participate. The aims of this process are simple but substantial: to encourage and inform the global and regional policies and practices necessary for bioenergy to play a prominent role in a sustainable future.

## 4. Interim recommendations

Informed by the five GSB conventions, we offer the following recommendations for productively approaching the bioenergy field and policy development:

— Realize that it may be more productive, and also more correct, to view the seemingly divergent assessments of bioenergy as answers to two different questions rather than the same question. Viewed in this light, there is considerably more scope for reconciliation than might first be apparent, and it is possible to be informed rather than paralysed by divergent assessments.
— Develop established and advanced bioenergy technologies such that each contributes to the other's success. That is, support and deploy in the near-term meritorious, established technologies in ways that enhance rather than impede deployment of advanced technologies, and support and deploy advanced technologies in ways that expand rather than contract opportunities for early adopters and investors.
— Be clear in formulating policies what mix of objectives are being targeted, measure the results of these policies against these objectives, and beware of unintended consequences.
— Undertake further exploration of land efficiency levers and visions for multiply-beneficial bioenergy deployment. This should be unconstrained by current practices, since we cannot hope to achieve a sustainable and secure future by continuing the practices that have led to the unsustainable and insecure present. It should also be approached from a global perspective, based on the best science available, and consider the diverse realities, constraints, needs and opportunities extant in different regions of the world.

## Footnotes

1 Total biomass energy in 2006 was 49.7 EJ of which 30.3 EJ was traditional biomass and 19.4 EJ was modern biomass. These values may be compared with total primary energy demand of 492 EJ [9].
